# Supplementary figures and images for: Genome-wide analyses of the bHLH gene family reveals structural and functional characteristics in the aquatic plant Nelumbo nucifera
Source: PeerJ. 2019 Jun 14;7:e7153. doi: 10.7717/peerj.7153 (PMC6573809; doi:10.7717/peerj.7153)

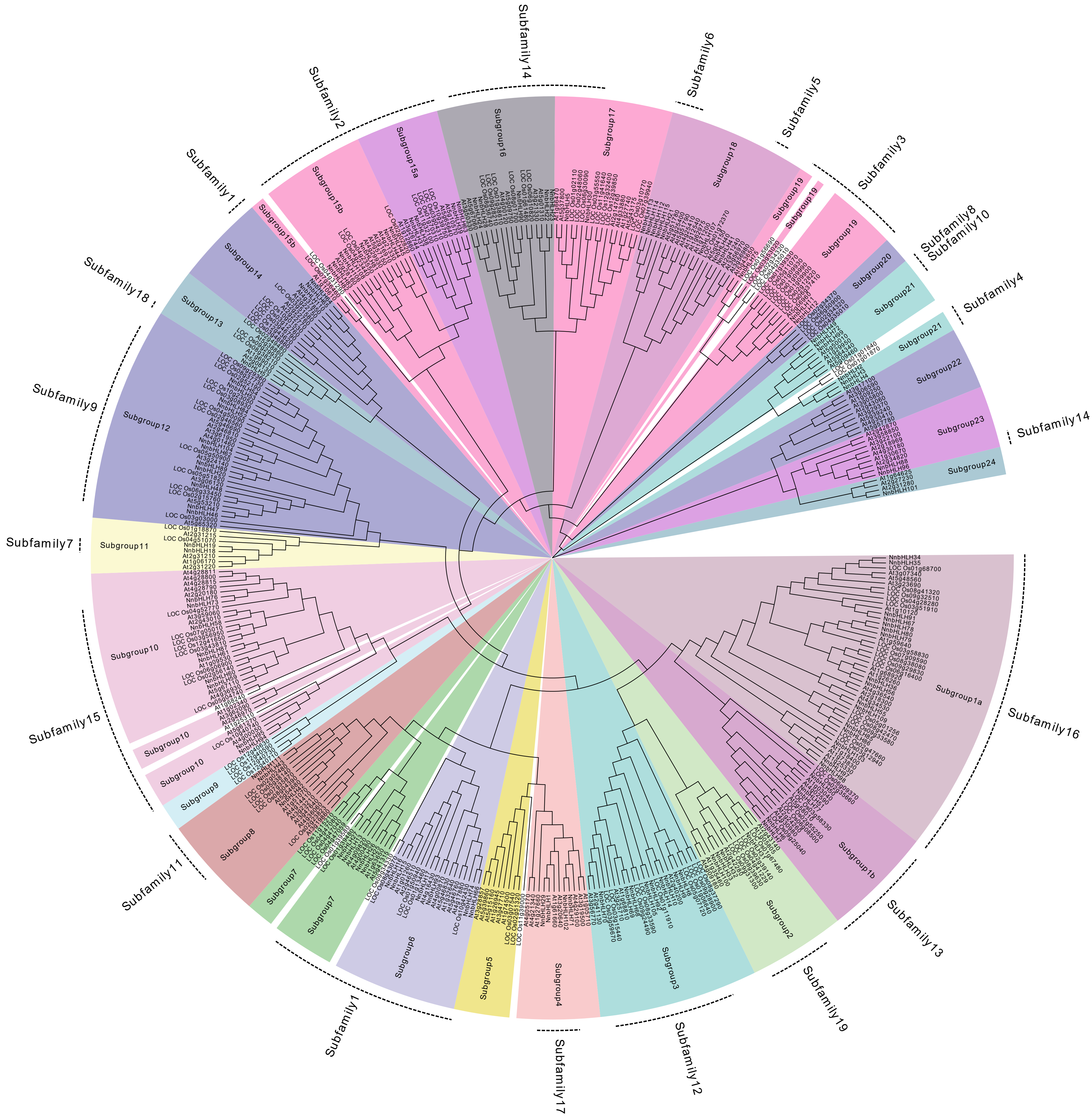

Supplement: Supplemental Information 2 [file peerj-07-7153-s002.pdf]
